# Incidence and Prevalence Trends of Pediatric Inflammatory Bowel Disease in the Daegu-Kyungpook Province From 2017 to 2020

**DOI:** 10.3389/fped.2021.810173

**Published:** 2022-01-04

**Authors:** Jae Young Choe, Sujin Choi, Ki Hwan Song, Hyo-Jeong Jang, Kwang-Hae Choi, Dae Yong Yi, Suk Jin Hong, Jun Hyun Hwang, Seung-Man Cho, Young Jin Kim, Byung-Ho Choe, Ben Kang

**Affiliations:** ^1^Department of Pediatrics, School of Medicine, Kyungpook National University, Daegu, South Korea; ^2^Department of Emergency Medicine, School of Medicine, Kyungpook National University, Daegu, South Korea; ^3^Department of Surgery, Goo Hospital, Daegu, South Korea; ^4^Department of Pediatrics, Keimyung University School of Medicine, Daegu, South Korea; ^5^Department of Pediatrics, Yeungnam University School of Medicine, Daegu, South Korea; ^6^Department of Pediatrics, College of Medicine, Chung-Ang University, Seoul, South Korea; ^7^Department of Pediatrics, Daegu Catholic University School of Medicine, Daegu, South Korea; ^8^Department of Preventive Medicine, Daegu Catholic University School of Medicine, Daegu, South Korea; ^9^Department of Pediatrics, Dongguk University School of Medicine, Gyeongju, South Korea; ^10^Department of Pediatrics, Daegu Fatima Hospital, Daegu, South Korea

**Keywords:** COVID-19, inflammatory bowel disease, Crohn's disease, Korea, Asia, child

## Abstract

**Background and Aim:** There is paucity of data regarding the epidemiology of pediatric IBD in Asia compared to that of Western countries. We aimed to investigate the incidence and prevalence trends of pediatric inflammatory bowel disease (IBD) in the Daegu-Kyungpook province of South Korea from 2017 to 2020.

**Methods:** This study was a multicenter, retrospective study conducted in eight IBD referral centers located in the Daegu-Kyungpook province. Children and adolescents of ≤18 years who were initially diagnosed with IBD between 2017 and 2020 were included. The annual number of children and adolescents newly diagnosed with IBD and the annual resident population of children and adolescents ≤18 years of age in the Daegu-Kyungpook province were investigated to calculate the annual incidence and prevalence in the region.

**Results:** A total 304 children and adolescents that had been diagnosed with IBD were included in this study. Among these patients, 71.4% had been diagnosed with Crohn's disease (CD), and 28.6% with ulcerative colitis (UC). The population based annual incidences of IBD from 2017 to 2020 were each 7.24, 6.82, 10.27, and 13.33 per 100,000, respectively (*P* for trend <0.001), 4.48, 5.26, 7.39, and 9.8 per 100,000, respectively, for CD (*P* for trend <0.001), and 2.76, 1.56, 2.88, and 3.53 per 100,000, respectively, for UC (*P* for trend = 0.174).

**Conclusion:** Pediatric IBD, especially CD has significantly increased recently in the Daegu-Kyungpook province. Epidemiology studies from other regions of Asia are required to better elucidate this trend of increase in Asia.

## Introduction

The prevalence and incidence of pediatric inflammatory bowel disease (IBD) have increased rapidly worldwide over the past two decades. As the pathophysiology of IBD is yet to be elucidated, it is important to understand the epidemiology of IBD to understand the genetic and environmental factors (1). While the incidence of adult IBD is stagnant in some Western countries, the incidence in Asian countries, such as Japan, Korea and Singapore, is still on a rise (2–5). This difference in incidence trends between countries according to geographical location in adults has also been observed in children with IBD (6–8).

Various changes have occurred in the incidence trends of pediatric diseases due to the recent coronavirus infectious disease 2019 (COVID-19) pandemic. The increase of childhood obesity and decrease of infection-related systemic diseases is associated with compliance with the quarantine rules and changes in living patterns including increased activities at home (9–12).

In this study, we aimed to investigate the incidence and prevalence trends of pediatric IBD from 2017 to 2020 in the Daegu-Kyungpook province of South Korea.

## Materials and Methods

### Patients and Study Design

**Figure 1:**
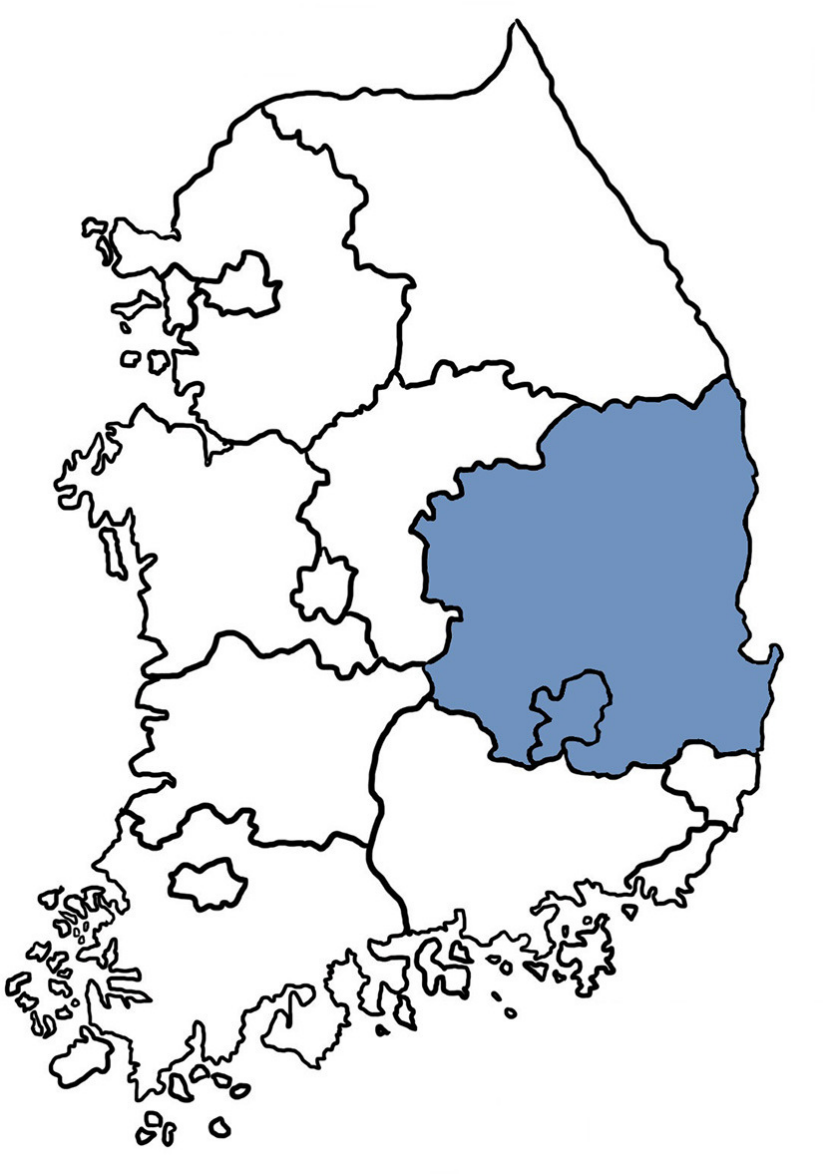
Map of South Korea and the Daegu-Kyungpook province.

This study was a multicenter, retrospective study conducted in eight IBD referral centers located in the Daegu-Kyungpook province: Kyungpook National University Children's Hospital affiliated with Kyungpook National University Chilgok Hospital, Keimyung University Dongsan Medical Center, Yeungnam University Medical Center, Kyungpook National University Hospital, Daegu Catholic University Medical Center, Dongguk University Gyeongju Hospital, Koo Hospital, and Daegu Fatima Hospital. These eight centers are the only pediatric IBD referral centers in the Daegu-Kyungpook province (Figure 1). Children and adolescents of ≤18 years who were initially diagnosed with IBD between January 1, 2017 and December 31, 2020 were included.

Baseline demographics including gender, birth date, diagnosis date, diagnosis age, and type of IBD were retrieved from electronic medical charts by searching for diagnosis codes of 555.X and K50.XX for CD and 556.X and K51.XX for UC according to the ICD-9 and ICD-10 codes, respectively. Patients residing outside the Daegu-Kyungpook province at diagnosis were excluded from the incidence analysis, and those who had moved outside the Daegu-Kyungpook province were excluded from the next particular year of the prevalence analysis.

The annual number of children and adolescents newly diagnosed with IBD at the centers and the annual resident population of children and adolescents ≤18 years in the Daegu-Kyungpook province were investigated to calculate the population based annual incidence and prevalence of pediatric IBD in the region. By utilizing data from the Statistics Korea which is a government organization for statistics under the Ministry of Economy and Finance, the population based per 100,000 incidence and prevalence of IBD, Crohn's disease (CD), and ulcerative colitis (UC) were each calculated, respectively.

In addition, annual trends were analyzed according to the type of disease, gender, and age groups according to the Paris classification (13). Comparison of demographic factors was also conducted between patients diagnosed in the pre-COVID-19 (2017–2019) and the post-COVID-19 era (2020).

### Statistical Analysis

For statistical comparison between two groups, chi square test or Fisher's exact test was used for categorical variables, while Student's *t*-test were used for statistical comparison of continuous variables. Comparative data for continuous variables are expressed as medians with interquartile range (IQR) or means with standard deviation (SD). To test the statistical significance of the fluctuations of annual incidence, 95% confidence intervals (CIs) calculated by the Clopper-Pearson method (14) for incidence were compared among years. The Cochran-Armitage test was used to evaluate the incidence trend of IBD. Data were considered to be statistically significantly different if *P* < 0.05. Statistical analyses were conducted using R version 3.2.3 (http://www.r-project.org).

### Ethics Statement

This study was conducted with approval from the Institutional Review Board (IRB) of Daegu Joint (IRB No. 2021-02-023), and informed consent was waived due to the retrospective nature of the study. We conducted this study in compliance with the principles of the Declaration of Helsinki.

## Results

### Baseline Characteristics

A total of 304 incidence cases and 1,065 prevalence cases were analyzed in this study. Of the 304 incidence cases, 217 were diagnosed with CD (71.4%) and 87 were diagnosed with UC (28.6%). The mean ratio of CD to UC was 2.5:1. There were 150 males, and 67 females for CD, and 59 males and 28 females for UC. The ratio of male to female was 2.2:1. The mean ± SD age at diagnosis was 14.6 ± 3.4 for CD and 15.3 ± 4.1 for UC.

### Incidence Trend of Pediatric IBD From 2017 to 2020

Annual incidences of IBD from 2017 to 2020 were each 63, 57, 82, and 102, respectively. Annual incidences of CD from 2017 to 2020 were each 39, 44, 59, and 75, respectively, while that of UC were each 24, 13, 23, and 27, respectively. Comparison of demographic factors between years are shown in Table 1. The annual number of newly diagnosed cases divided according to the Paris classification age is shown in Table 2.

**Table 1:** Annual newly diagnosed cases, population, and comparison of demographic factors between years.

**Demographic factors**	**2017**	**2018**	**2019**	**2020**	***P***
IBD	63	57	82	102	NA
CD	39	44	59	75	NA
UC	24	13	23	27	NA
Population, *n*	869,801	835,932	798,713	765,440	NA
CD:UC ratio	1.6:1	3.4:1	2.6:1	2.8:1	0.266
M:F ratio	1.7:1	2.4:1	2.4:1	2.3:1	0.789
Diagnosis age, *year* (IQR)	15.0 (13.1–17.5)	15.7 (14.2–17.4)	16.5 (13.5–17.8)	15.4 (13.4–17.2)	0.424

*IBD, inflammatory bowel disease; CD, Crohn's disease; UC, ulcerative colitis*.

**Table 2:** Annually newly diagnosed cases according to the Paris classification age.

**Paris classification age, *n* (%)**	**2017**	**2018**	**2019**	**2020**
**IBD**				
0–9	12 (35.3)	4 (11.8)	10 (29.4)	8 (23.5)
10–16	31 (18.8)	35 (21.2)	37 (22.4)	62 (37.6)
17–18	20 (19.1)	18 (17.1)	35 (33.3)	32 (30.5)
**CD**				
0–9	8 (32.0)	3 (12.0)	9 (36.0)	5 (20.0)
10–16	22 (17.2)	28 (21.9)	30 (23.4)	48 (37.5)
17–18	9 (14.0)	13 (20.3)	20 (31.3)	22 (34.4)
**UC**				
0–9	4 (44.4)	1 (11.1)	1 (11.1)	3 (33.3)
10–16	9 (24.4)	7 (18.9)	7 (18.9)	14 (37.8)
17–18	11(26.8)	5 (12.2)	15 (36.6)	10 (24.4)

*IBD, inflammatory bowel disease; CD, Crohn's disease; UC, ulcerative colitis*.

### Comparison Between the Pre-COVID-19 and Post-COVID-19 Era

Comparison was conducted between patients diagnosed in the pre-COVID-19 (2017–2019) and the post-COVID-19 era (2020). Comparison of demographic factors are shown in Table 3.

**Table 3:** Comparison of demographic factors between patients diagnosed in the pre- and post-COVID-19 era.

	**Pre-COVID-19 (*n* = 202)**	**Post-COVID-19 (*n* = 102)**	***P***
Diagnosis, *n* (%)			0.650
CD	142 (70.3)	75 (73.5)	
UC	60 (29.7)	27 (26.5)	
CD:UC ratio	2.4:1	2.8:1	0.650
Gender, *n* (%)			0.922
Male	138 (68.3)	71 (69.6)	
Female	64 (31.7)	31 (30.4)	
Male:Female ratio	2.2:1	2.3:1	0.922
Diagnosis age, year	15.8 (13.5–17.5)	15.4 (13.4–17.2)	0.346
Paris classification age, year			0.183
0–9	26 (12.9)	8 (7.8)	
10–16	102 (50.5)	62 (60.8)	
17–18	74 (36.6)	32 (31.4)	

*CD, Crohn's disease; UC, ulcerative colitis*.

**Figure 2:**
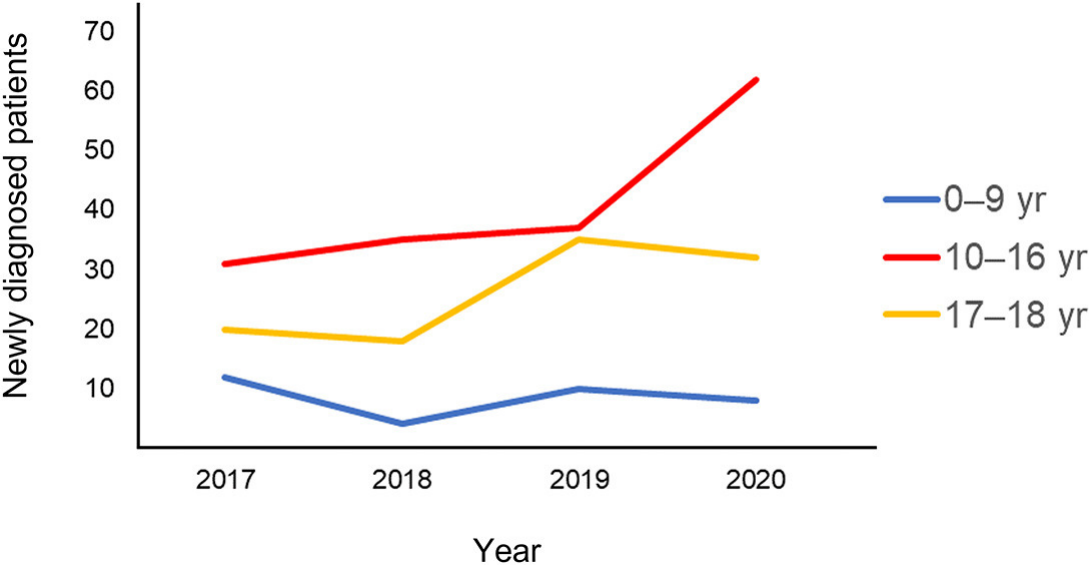
Annual changes in pediatric IBD, CD, and UC incidence according to age. IBD, inflammatory bowel disease; CD, Crohn's disease; UC, ulcerative colitis.

Compared to the pre-COVID-19 era, increases of incidences in the post-COVID-19 era for both pediatric CD and UC were observed only in the age group of 10–16 years (Figure 2). Although a statistical significance was not observed, the proportion of patients 10–16 years had increased from 50.5% in 2019 to 60.8% in 2020 among IBD patients (*P* = 0.183). The proportion of patients in the age group of 10–16 years had increased from 56.3% in 2019 to 64.0% in 2020 among patients diagnosed with CD (*P* = 0.243). The proportion of patients in the age group of 10–16 years had also increased from 36.7% in 2019 to 51.9% in 2020 among patients diagnosed with UC (*P* = 0.352).

### Population Based Incidence Trend of Pediatric IBD From 2017 to 2020

**Figure 3:**
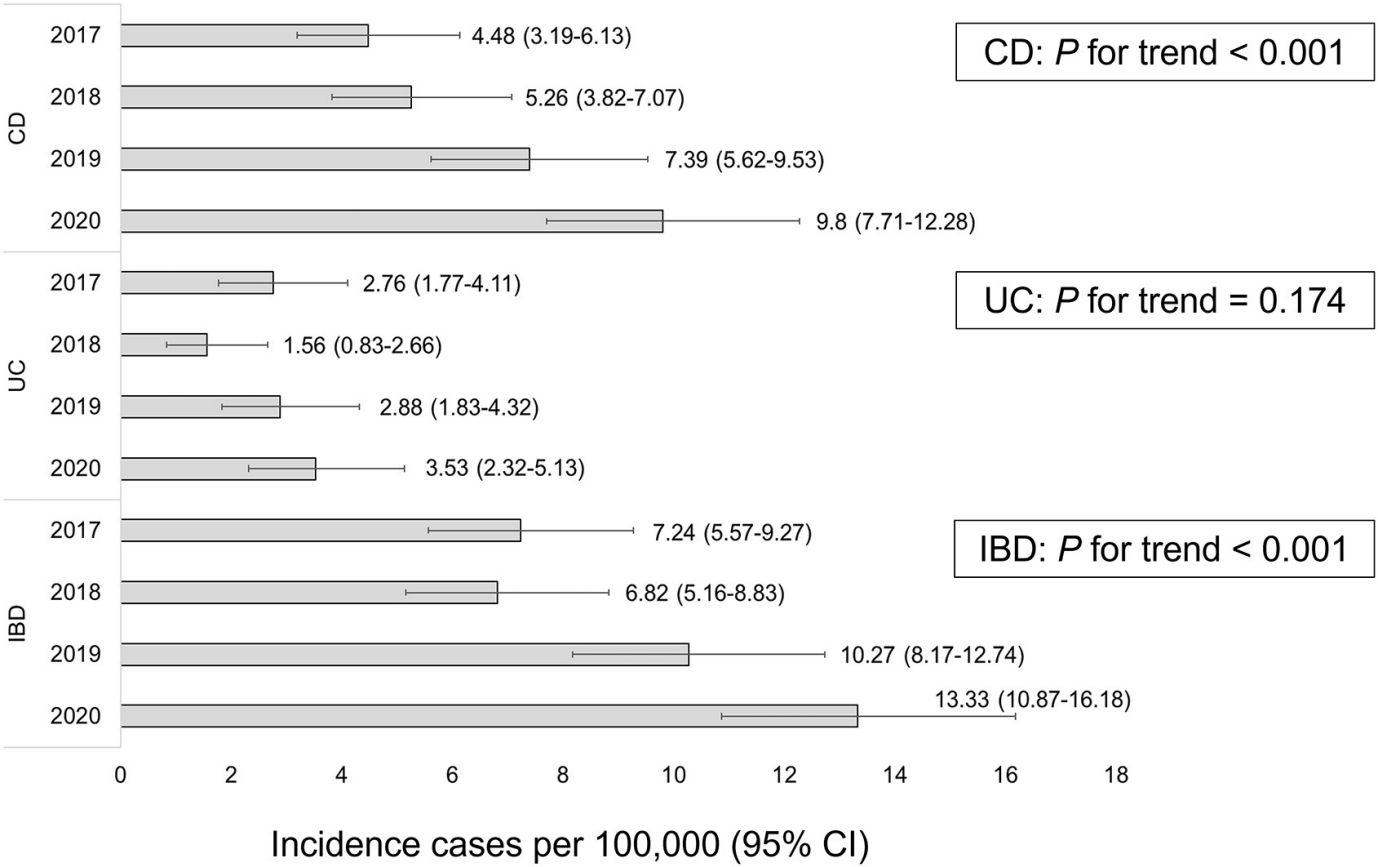
Population based incidence of pediatric CD, UC, and IBD per year. CD, Crohn's disease; UC, ulcerative colitis; IBD, inflammatory bowel disease.

**Figure 4:**
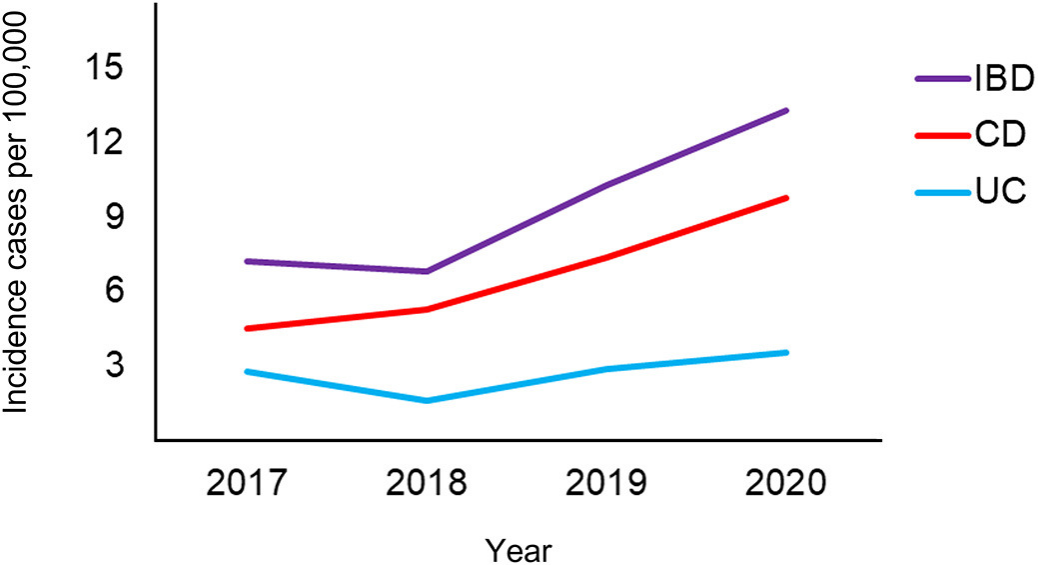
Annual changes in the population based incidence of pediatric IBD, CD, and UC. IBD, inflammatory bowel disease; CD, Crohn's disease; UC, ulcerative colitis.

The population based annual incidences of IBD from 2017 to 2020 were each 7.24, 6.82, 10.27, and 13.33 per 100,000, respectively (*P* for trend <0.001). The population based annual incidences of CD from 2017 to 2020 were each 4.48, 5.26, 7.39, and 9.8 per 100,000, respectively (*P* for trend <0.001). The population based annual incidences of UC from 2017 to 2020 were each 2.76, 1.56, 2.88, and 3.53 per 100,000, respectively (*P* for trend = 0.174) (Figure 3). Pediatric CD showed a continuous increase from 4.48 (95%, CI, 3.19–6.13) per 100,000 in 2017 to 9.8 (7.71–12.28) per 100,000 in 2020, but pediatric UC showed a temporary decrease to 1.56 (95%, CI, 1.83–4.32) in 2018, followed by a continuous increase to 3.53 (95%, CI, 2.32–5.13) in 2020 (Figure 4; Table 4).

**Table 4:** Annual incidence and population based annual incidence of pediatric IBD, CD, and UC.

	**2017**	**2018**	**2019**	**2020**
IBD	7.24 (5.57–9.27)	6.82 (5.16–8.83)	10.27 (8.17–12.74)	13.33 (10.87–16.18)
CD	4.48 (3.19–6.13)	5.26 (3.82–7.07)	7.39 (5.62–9.53)	9.8 (7.71–12.28)
UC	2.76 (1.77–4.11)	1.56 (1.83–4.32)	2.88 (1.83–4.32)	3.53 (2.32–5.13)

*IBD, inflammatory bowel disease; CD, Crohn's disease; UC, ulcerative colitis*.

### Population Based Prevalence Trend of Pediatric IBD From 2017 to 2020

The prevalence of pediatric IBD from 2017 to 2020 showed a steady increase, which was 28.3, 29.9, 32.8 and 40.1 per 100,000 each year respectively in the Daegu-Kyungpook province. The prevalence of pediatric CD was 21.4, 23.0, 25.5, and 30.8 per 100,000, while the prevalence of pediatric UC was 6.9, 6.9, 6.9, and 9.3 per 100,000 each year, respectively, from 2017 to 2020.

## Discussion

In this study, we identified a significant increase in the incidence and prevalence of pediatric IBD, CD, and UC, respectively, in the Daegu-Kyungpook province of South Korea from 2017 to 2020.

The metropolitan city of Daegu is the fourth largest city in Korea located in the southeast with a population of ~2.5 million (Figure 1). The combined population of Daegu-Kyungpook province accounts for 9.8% of the total Korean population as of 2020, and 15.1% of the total population of children under the age of 18 (15). Seven centers participating in this study are located in Daegu, while one center is located in the city of Gyeongju of Kyungpook province. These eight centers are the only referral centers capable of care for pediatric IBD patients in the Daegu-Kyungpook province. Therefore, it is likely that the incidence and prevalence in this study represents that of the general population in this age group in the region.

Unlike the plateau in pediatric IBD incidence in Western countries, the incidence of pediatric IBD is on a sharp rise in Asia (6, 8, 16). According to a recent systematic review, the incidence of IBD, CD, and UC were each 0.5–11.4, 0.3–3.7, and 0.2–3.9 per 100,000 person-years, respectively, from 1968 to 2012 (8). However, when compared to the adult data on IBD epidemiology, there are still very few studies from Asian countries with relatively high population such as China, India, and Central Asia (17–20). Owing to the limited data regarding pediatric IBD epidemiology in Asia, this study may be an important piece of the puzzle to define the epidemiology of pediatric IBD in the region.

CD to UC ratio may vary according to factors such as age, region, and also over time (2, 21). In a study conducted in Japan, pediatric UC dominated pediatric CD (22). Meanwhile, a study conducted in Singapore showed a CD dominance over UC in the pediatric age group, which is comparable of the results in this study (23). Globally, CD predominates over UC in regions of high IBD incidence (8). Recent data have shown higher rates of pediatric CD than UC in Europe and North America (8). Meanwhile, similar to the ratio of Japan, UC incidence is higher than that of CD in northern California, Finland, and Italy (24–26). The reason for these differences between regions is yet to be elucidated.

In our previous study that investigated the incidence of pediatric IBD from 2011 to 2016 in the Daegu-Kyungpook province, mean pediatric CD to UC ratio was 4.1:1 (27). However, in this study, mean pediatric CD to UC ratio was reduced to 2.5:1. Compared to our previous study, the annual number of pediatric CD cases increased 3.3-fold, while the number of pediatric UC cases showed a sharp increase of 5.4-fold. Thus, the relatively higher increase rate in pediatric UC cases compared to CD cases may have contributed to this result.

Several studies have reported that the age at diagnosis of IBD is decreasing with time (28–32). A recent study conducted in Israel showed that there was an average decrease in age at diagnosis from 15.0 ± 2.8 in 2002–2008 to 14.3 ± 3.1 in 2009–2016 (*P* < 0.001). Although we were unable to identify such statistically significant difference due to the short study period, we were able to observe that the median diagnosis age in 2020 had decreased compared to 2019. The decrease in diagnosis age may hypothetically result from the change in disease biology, known as the concept of genetic anticipation (30). Although genetic anticipation is well-established in some monogenic disorders, there are controversies regarding its relevance to polygenic disorders, such as IBD (30).

Although a statically significant increase was not identified compared to the pre-COVID-19 era, the highest annual incidences for pediatric IBD, CD and UC in this study were all observed in the post-COVID-19 era of 2020. Although there have been studies investigating the changes incidence of other diseases or health conditions (9–12), no study in Asia has yet investigated the change of incidence in pediatric IBD patients after the COVID-19 pandemic. This study is the first in Asia to investigate the incidence in the pediatric population of IBD after the COVID-19 pandemic.

The increase of pediatric IBD incidence in the Daegu-Kyungpook province in the post-COVID-19 era may be attributable to several reasons. First, the increase may be merely on the same line of a continuous increase of pediatric IBD in Korea. The increase of pediatric IBD has been reported worldwide, especially in Asian countries (6, 19, 22, 23, 31). There have also been studies on the recent rapid increase in pediatric IBD incidence in Korea (27, 32, 33). Pediatric IBD incidences as high as 14.3–18.3 per 100,000 have been reported in regions from Western countries (34–38). Although the incidence of IBD remains highest in the northern populations of Europe and America, it has remained stable or even decreased (8). To our knowledge, the pediatric IBD incidence of 13.3 per 100,000 as of 2020 in this study is the highest in Asia, and shows that the incidence in the Daegu-Kyungpook province has nearly reached that of the highest regions in the world.

Second, the relative increase of pediatric IBD incidence I n the Daegu-Kyungpook province may be a result of a sharp decrease in the number of children in the region. During the study period, the total population in Daegu-Kyungpook province decreased by 1.1% from 5,133,000 to 5,074,000, while the number of children under the age of 18 decreased by 12.0% from 869,801 to 765,440 (14). As of 2020, Korea has the lowest birth rate in the world with a total fertility rate of 0.84 (39). The birth rate in Daegu-Kyungpook province is also continuously decreasing.

Third, the change in lifestyle in the post-COVID-19 era may have influenced this increase. The COVID-19 pandemic has led to increased time spent at home and reduced physical activity, which has caused an increase in childhood obesity (9). In addition, the increased time spent at home and the explosion of food delivery services have increased the frequency of exposure to processed food instead of homemade food. This change in eating habits and activity may have led to an increase of exposure to environmental risk factors. Changes in diet and consequent alteration in the composition of the intestinal microbiota may be associated with the increasing incidence of pediatric IBD (40).

There are some limitations of this study, and therefore results of this study should be interpretated with caution. First, this study was conducted in a particular region in Korea, and therefore the results may not represent that of the whole country. Second, there is a possibility that some pediatric IBD patients in the region follow up to centers outside the region. Therefore, there is a possibility that these patients were excluded from this analysis. However, because most patients are initially diagnosed at centers located at the administrative district of residence, we believe the number of these patients are extremely small to influence the results of this study. Third, 4 years may be rather short for investigating the incidence trend in a particular region. Fourth, we were unable to investigate the incidence of IBD-unclassified (IBD-U) due to the lack of a diagnostic code for IBD-U on ICD-9 and ICD-10. Last but not least, this study was a retrospective study.

In conclusion, we identified a significant increase in the incidence and prevalence of pediatric IBD in the Daegu-Kyungpook province of South Korea from 2017 to 2020. Unlike the decrease in pediatric infectious diseases or pediatric infection-related systemic diseases in the post-COVID-19 era, pediatric IBD has significantly increased in the Daegu-Kyungpook province. Epidemiology studies from other regions are required to better identify whether the COVID-19 pandemic has influenced pediatric IBD incidence or not.

## Data Availability Statement

The raw data supporting the conclusions of this article will be made available by the authors, without undue reservation.

## Ethics Statement

The studies involving human participants were reviewed and approved by Institutional Review Board (IRB) of Daegu Joint (IRB No. 2021-02-023). Written informed consent from the participants' legal guardian/next of kin was not required to participate in this study in accordance with the national legislation and the institutional requirements.

## Author Contributions

JC, SC, H-JJ, SH, and S-MC contributed in the acquisition, analysis and interpretation of data, and drafting of the initial manuscript. KS, K-HC, DY, JH, YK, and B-HC contributed in the acquisition, analysis and interpretation of data, and critical revision for important intellectual content. BK contributed in the conception of the study, acquisition, analysis and interpretation of data, drafting of the initial manuscript, and critical revision for important intellectual content. All authors approved the final version of the manuscript and agreed to be accountable for all aspects of the work.

## Funding

This work was supported by the National Research Foundation of Korea (NRF) grant funded by the Korean government (MSIT) (No. 2021R1A2C1011004).

## Conflict of Interest

The authors declare that the research was conducted in the absence of any commercial or financial relationships that could be construed as a potential conflict of interest.

## Publisher's Note

All claims expressed in this article are solely those of the authors and do not necessarily represent those of their affiliated organizations, or those of the publisher, the editors and the reviewers. Any product that may be evaluated in this article, or claim that may be made by its manufacturer, is not guaranteed or endorsed by the publisher.
